# Letter to the Editor: Stroke volume is the key measure of fluid responsiveness

**DOI:** 10.1186/s13054-021-03498-5

**Published:** 2021-03-15

**Authors:** Jon-Emile S. Kenny, Igor Barjaktarevic

**Affiliations:** 1 Health Sciences North Research Institute, 56 Walford Rd, Sudbury, ON P3E 2H2 Canada; 2 Division of Pulmonary and Critical Care, Department of Medicine, David Geffen School of Medicine At UCLA, Los Angeles, CA USA

## To the Editor

The authorities in the domain of shock physiology, Vincent and colleagues, have recently offered a concise perspective on intravenous fluid administration [1]. They discuss the concept of the fluid challenge and offer a practical clinical algorithm, but miss an opportunity to underscore that cardiac output (CO) is a complex measure when interpreting the full effect of fluid provision.

Historically, fluid responsiveness (FR) has been defined by change in cardiac output (CO = heart rate x stroke volume) as CO is a key determinant of oxygen delivery [1, 2]. Ignoring issues around measurement precision and defining a true gold standard, we note that relying solely on CO belies the fundamental physiology that *stroke volume* is the closest clinically-available approximation of the cardiac length-tension relationship. This point is highlighted by a hypothetical example.

A patient in early septic shock has a heart rate (HR) of 120 beats per minute (BPM), stroke volume (SV) of 51 mL and, therefore, a CO of 6.1 L per minute (L/min). The patient is given 30 mL/kg of balanced crystalloid; subsequently HR falls to 90 BPM and SV rises to 65 mL resulting in a CO of 5.9 L/min. Was this patient harmed by intravenous fluids? Is this patient ‘fluid unresponsive’ despite SV rising by over 25%?

An implicit, mathematical assumption of CO is that its augmentation is beneficial independent of the relationship between SV and HR; this may not be universally true. For example, the coronary circulation is perfused preferentially during diastole. Therefore, diminishing HR augments diastolic time and subendocardial perfusion [3]. Accordingly, despite a calculated *fall* in cardiac output, the patient above may have significantly *increased* ventricular oxygen delivery with intravenous fluids, especially if there is co-morbid coronary artery disease and/or ventricular hypertrophy. These principles could partly explain recent data associating diminished diastolic shock index (i.e., HR divided by diastolic blood pressure) with improved outcome [4]. In other words, *low* HR and high diastolic pressure is a good prognosticator in septic shock.

Thus, because CO comprises two variables that may diverge as a normal, adaptive response or following beneficial therapy, relying on it as a lumped index of ‘fluid responsiveness’ may mislead. Accordingly, clinicians and researchers should rely not merely on CO as it may conceal the effect of fluids on the cardiac length-tension, i.e., Frank-Starling, relationship.

## Data Availability

Not Applicable
