# Supplementary material for: Letter to the Editor: Stroke volume is the key measure of fluid responsiveness
Source: Crit Care. 2021 Mar 15;25:104. doi: 10.1186/s13054-021-03498-5 (PMC7962206; doi:10.1186/s13054-021-03498-5)
Supplement: Related texts

# Reply: Authors’ response

**Authors:** Maurizio Cecconi, Daniel De Backer, Jean-Louis Vincent

**Affiliations:** 3 grid.417728.f 0000 0004 1756 8807 Department of Anesthesiology and Intensive Care, Humanitas Clinical and Research Center-IRCCS, Rozzano, Milan Italy; 4 grid.452490.e Department of Biomedical Sciences, Humanitas University, Pieve Emanuele, Milan, Italy; 5 grid.4989.c 0000 0001 2348 0746 Department of Intensive Care, CHIREC Hospital, Université Libre de Bruxelles, Brussels, Belgium; 6 Department of Intensive Care, Route de Lennik 808, Erasme Hospital, Université Libre de Bruxelles, 1070 Brussels, Belgium

## Stroke volume versus cardiac output during a fluid challenge

We would like to thank Drs Kenny and Barjaktarevic for their comment about our paper on fluid challenge [1]. The authors are correct to indicate that stroke volume (SV) should be the preferred index for evaluating fluid responsiveness, rather than cardiac output (CO). As indicted in the example provided by Kenny and Barjaktarevic, a patient can respond to fluids by increasing SV and decreasing heart rate, so that CO may not increase. However, if the goal of fluid administration is to increase oxygen delivery (DO_2_), this will not be achieved by increasing SV without an increase in CO. A decrease in hemoglobin concentration due to a dilutional effect of a large bolus of fluid may also limit the increase in DO_2_.

Measurements of CO rather than SV also have some practical advantages. First, the decrease in heart rate can vary during a fluid challenge. In a study evaluating fluid responsiveness in 491 critically ill patients [5], heart rate decreased minimally in fluid responders and non-responders, and the area under the receiver-operating characteristic curve for changes in heart rate to detect fluid response was only 0.53, i.e., hardly better than flipping a coin. Second, measuring SV is challenging in fluid responders who may have respiratory variations in SV that decrease as fluid is administered [6]. As suggested by Kenny and Barjaktarevic, in some cases, a reduction in heart rate may protect coronary perfusion by prolonging diastole, but prolonging diastole can also decrease diastolic pressure. Increasing vasopressor support may be a better option to increase coronary perfusion [7].

Perhaps, the most important message is that the management of critically ill patients is complex and cannot be based on one variable only. Using SV to assess fluid response is not wrong if one looks at the Frank–Starling relationship, but ultimately the DO_2_ is what matters for the tissues.
